# Characterization of Human Norovirus Nonstructural Protein NS1.2 Involved in the Induction of the Filamentous Endoplasmic Reticulum, Enlarged Lipid Droplets, LC3 Recruitment, and Interaction with NTPase and NS4

**DOI:** 10.3390/v15030812

**Published:** 2023-03-22

**Authors:** Chien-Hui Hung, Ju-Bei Yen, Pey-Jium Chang, Lee-Wen Chen, Tsung-Yu Huang, Wan-Ju Tsai, Yu-Chin Tsai

**Affiliations:** 1 Graduate Institute of Clinical Medical Sciences, Chang Gung University, Taoyuan 33302, Taiwan; peyjiumc@mail.cgu.edu.tw (P.-J.C.);; 2 Department of Internal Medicine, Division of Infectious Diseases, Chang Gung Memorial Hospital, Chiayi 61363, Taiwan; 3 Department of Pediatrics, Chang Gung Memorial Hospital, Chiayi 61363, Taiwan; coner@adm.cgmh.org.tw; 4 Department of Respiratory Care, Chung Gung University of Science and Technology, Chiayi 61363, Taiwan

**Keywords:** norovirus, NS1.2, LC3, autophagy-independent pathway, NTPase, NS4, viral replication complex

## Abstract

Human noroviruses (HuNVs) are the leading cause of gastroenteritis worldwide. NS1.2 is critical for HuNV pathogenesis, but the function is still unclear. The GII NS1.2 of HuNVs, unlike GI NS1.2, was localized to the endoplasmic reticulum (ER) and lipid droplets (LDs) and is accompanied by a distorted-filamentous ER morphology and aggregated-enlarged LDs. LC3 was recruited to the NS1.2-localized membrane through an autophagy-independent pathway. NS1.2, expressed from a cDNA clone of GII.4 norovirus, formed complexes with NTPase and NS4, which exhibited aggregated vesicle-like structures that were also colocalized with LC3 and LDs. NS1.2 is structurally divided into three domains from the N terminus: an inherently disordered region (IDR), a region that contains a putative hydrolase with the H-box/NC catalytic center (H-box/NC), and a C-terminal 251–330 a.a. region containing membrane-targeting domain. All three functional domains of NS1.2 were required for the induction of the filamentous ER. The IDR was essential for LC3 recruitment by NS1.2. Both the H-Box/NC and membrane-targeting domains are required for the induction of aggregated-enlarged LDs, NS1.2 self-assembly, and interaction with NTPase. The membrane-targeting domain was sufficient to interact with NS4. The study characterized the NS1.2 domain required for membrane targeting and protein–protein interactions, which are crucial for forming a viral replication complex.

## 1. Introduction

Human norovirus (HuNoV) is the leading cause of gastroenteritis outbreaks globally [1] and is spread through contaminated water or food and person-to-person transmission [2,3]. HuNoV infects approximately 685 million individuals worldwide annually [4]. Despite the considerable impact of HuNoV, no commercial vaccines or specific antiviral drugs are available to prevent HuNoV infections. HuNoV belongs to the genus *Norovirus* in the family *Caliciviridae* [5] and is subdivided into at least ten genogroups (GI–GX) [6]. GI, GII, GIV, GVIII, and GIX infect humans and can be further classified into various genotypes [6]. Most of the reported norovirus outbreaks during the past decade were caused by the norovirus GII.4 genotype [7,8]. The norovirus GII.17 variant has recently emerged as a dominant strain in East Asia [9].

HuNoV is a positive-sense single-stranded RNA virus. Its genome is approximately 7.5 kb and covalently linked to a virus-encoded nonstructural protein (VPg) at the 5′ end and polyadenylated at the 3′ end [10]. The genome contains three open reading frames (ORFs). ORF1 encodes a large polyprotein that is post- and co-translationally cleaved by a virus-encoded protease (NS6), producing six nonstructural proteins: NS1.2, NTPase (NS3), NS4, VPg (NS5), protease (Pro, NS6), and RNA-dependent RNA polymerase (RdRp, NS7). ORF2 encodes the major capsid protein VP1, and ORF3 encodes the minor capsid protein VP2 [11]. Because of the lack of a reverse genetic system to propagate HuNoV robustly and a small animal model system, HuNoV remains among the most poorly characterized groups of RNA viruses. Although in vitro culture systems have been developed using B cells and human intestinal enteroids recently [12,13], the replication system is limited by the use of clinically isolated fecal samples. Viral replication and pathogenicity mechanisms remain to be determined. Features of the lifecycle of HuNoV have been inferred from studies on other animal caliciviruses that can be cultivated in mammalian cell cultures and the animal system (e.g., murine norovirus [MNV]) [14,15,16].

Similar to other positive-stranded RNA viruses, norovirus remodels the intracellular membrane to form a specialized viral replication compartment, termed as the replication complex, where viral RNA genome replication occurs [17,18]. The replication complex provides an optimal microenvironment for viral replication by concentrating viral components and host factors and providing a barrier that shields viral replication intermediates (e.g., dsRNA) from the host innate immune sensor [19]. Therefore, membrane remodeling is crucial in the lifecycle of positive-stranded RNA viruses. Understanding both the viral and host factors involved in the formation of the viral replication complex can facilitate the development of novel therapeutics. The replication complex of MNVs was determined to be associated with membrane vesicles derived from a secretory pathway [20]. Expressing ORF1 polyproteins of HuNoV is adequate to induce membrane alterations for forming a replication complex-like compartment similar to that observed in MNV-infected cells; this compartment consists of vesicles likely derived from the endoplasmic reticulum (ER). Furthermore, the nonstructural proteins NS1.2, NTPase, and NS4 contribute to viral membrane alterations [21] and are crucial for forming the viral replication complex.

The NS1.2 protein, located at the N terminus of the ORF1 polyprotein, is the only norovirus protein lacking a sequence homology with any other viral protein [22]. A comparison of proteins in databases revealed that NS1.2 contains putative H-box and NC motifs that form a catalytic domain of the NlpC/P60 superfamily of a circularly permutated enzyme [23]. The NS1.2 protein is composed of three domains: the N terminal of NS1.2 contains a highly disordered proline-rich domain (IDR) [24], a putative hydrolase contains the H-box/NC catalytic center (H-box/NC) in the middle [23], and a region, 251–330 a.a., contained predicted transmembrane hydrophobic region (amino acids 291–322) in the C terminus [25]. Cellular expression of MNV and feline calicivirus NS1.2 is localized to the ER [20,26], whereas HuNoV GI NS1.2 appears to be localized to the Golgi apparatus and causes Golgi disassembly [25]. GII.4 NS.1.2 localizes to the ER and induces the proliferation of smooth ER membranes, forming long tubular structures [21]. GI NS1.2 forms a complex with SNARE regulator VAP-A and disrupts intracellular vesicular trafficking [27]. Studies on MNV have reported that NS1.2 is cleaved by caspase during infection, separating NS1 from membrane-associated NS2 [28,29,30]. NS1 is secreted through an unconventional secretory pathway [28]. The *NS1* gene from the persistent MNV strain CW6, but not the acute strain CW3, promotes intestinal epithelial cell infection and causes persistent fecal shedding [29,30] by counteracting IFN-λ-mediated antiviral immunity [31,32]. In addition, vaccination with NS1 alone confers protection against MNV infection [32].

Membrane remodeling is the hallmark of positive-strand RNA viruses, including norovirus. NS1.2 is crucial for membrane alteration, but how the NS1.2 induced membrane alteration has not been fully explored. This study determined the domains required for the targeting of the ER or lipid droplet (LD) membrane, induction of the filamentous ER and enlarged LDs, recruitment of autophagy-independent LC3, self-assembly, and interacts with NTPase and NS4, which is crucial for the formation of the viral replication complex.

## 2. Materials and Methods

### 2.1. Cell Lines

HEK293T (ATCC CRL-3216), HeLa cells, wild-type mouse embryonic fibroblast (MEF) (RIKEN BRC2710), and Atg5 knockout MEF (RIKEN BRC2711) were maintained in DMEM supplemented with 10% fetal bovine serum (FBS). A7 (ATCC CRL-2500), a melanoma cell line, was cultured in MEM supplemented with 10% FBS and 400 μg/mL G418. AD293(GFP-LC3), an AD293 (Agilent Technologies, Santa Clara, CA, USA) cell stably expressed GFP-LC3, was cultured in DMEM containing 10% FBS and 400 μg/mL G418.

### 2.2. Plasmids

Plasmids pFlag-NS1.2, 3xMyc-tagged NS1.2, NTPase, NS4, protease, Vpg, and RdRp were constructed as described [33]. The cDNA of the GII.4 strain, HuNV/GII.4/YJB1/2009/Chiayi (GeneBank accession numbers is MG049692) [33], was cloned to pFlag-CMV2 to generate pFlag-NV. The deletion mutants of Flag-tagged NS1.2, NS1.2 (1–131), (117–250), (251–330), (233–330), (1–250), (1–280), (1–280L_262_L_266/_ED), (117–330), and (Δ117–250) were generated by using Q5 site-directed mutagenesis kit (NEB) using pFlag-NS1.2 as a template. The C-terminal Myc-tagged NS1.2 (1–250) and (233–330) were generated by using a Q5 site-directed mutagenesis kit (NEB) using Myc-tagged NS1.2 as a template. The C-terminal Myc-tagged NS1.2 (117–250) was generated by using a Q5 site-directed mutagenesis kit (NEB) using Myc-tagged NS1.2 (1–250) as a template. The GFP-NS1.2, GFP-NS1.2 (251–330), and GFP-NS1.2 (280–330) were generated by PCR amplified full-length NS1.2, or NS1.2 (251–330), or NS1.2 (280–330) and cloned into peGFP-C1, respectively. The GFP-NS1.2 (251–290) was generated by using Q5 site-directed mutagenesis kit using GFP-NS1.2 (251–330) as a template. The primers used in this study were listed in Table S1 of Supplementary Materials.

### 2.3. Immunoprecipitation and Immunoblot Analysis

Cells were lysed in PBS that contained 1% Triton X-100 and a protease inhibitor cocktail (Sigma Aldrich, St. Louis, MO, USA). Cell lysates were then cleared by centrifugation at 12,000× *g* at 4 °C for 15 min. Alternatively, cells were directly lysed in an electrophoresis sample buffer. For coimmunoprecipitation, GFP-trap and Myc-trap (ChromoTek GmbH, Munich, Germany) or Flag M2 conjugated magnetic beads (Sigma) were added, and the mixture was incubated at 4 °C for 2.5 h. After washing with PBS containing 1% Triton X-100, proteins bound to the beads were eluted by adding 2× electrophoresis sample buffer and analyzed by immunoblotting. The primary antibodies used in the study were as follows: Rabbit and mouse anti-Flag (Sigma Aldrich), mouse anti-Myc (Cell Signaling, Danvers, MA, USA), rabbit anti-Myc (MBL, Tokyo, Japan), mouse anti-GFP (Roche, Basel, Switzerland), mouse anti-GFP (Santa Cruz, Dallas, TX, USA), rabbit anti-NS1.2, anti-NTPase, and anti-NS4. Rabbit anti-NS1.2 was produced in this study immunized rabbit with NS1.2 (1–202), which was purified from bacteria expressed His-tagged NS1.2 (1–202) (AllBio Science Incorporated, Taichung Taiwan). Anti-NTPase and NS4 were kindly provided by Dr. Pey-Jium Chang [33].

### 2.4. Immunofluorescence Staining

Cells were fixed with 4% paraformaldehyde and processed for indirect immunofluorescence staining. Fixed cells were then permeabilized and blocked by incubating in PBS containing 0.5% Triton X-100 and 2% Normal Goat Serum (Sigma Aldrich) for 15 min at room temperature. After washing with PBS containing 0.1% Triton X-100 and 0.2% BSA, cells were conducted with primary antibodies as indicated following incubation with fluorescently labeled secondary antibodies (Invitrogen, Thermo Fisher Scientific, Waltham, MA, USA). Primary antibodies used in this study are as follows: anti-PDI, GM130 (BD Biosciences, Franklin Lakes, NJ, USA), mouse and rabbit anti-Flag (Sigma Aldrich), anti-Myc (Cell Signaling), anti-LC3 (Nanotools, Teningen, Germany), anti-NS1.2 (this study), anti-NTPAse, and anti-NS4 [33]. LipidTox red neutral lipid stain (Invitrogen) was used to stain lipid droplets in fixed cells. Images were captured using a Leica SP5 fluorescence microscope. The representative images in different fields were chosen and captured from more than 100 cells. The images shown are representative of experiments carried out at least three times.

### 2.5. Quantification of Lipid Droplets

For microscopy data collection, the images were randomly chosen. The sizes and numbers of LDs were determined using ImageJ software 1.53t (NIH, Bethesda, Maryland, USA). Single-channel images were exported, and the scale was set by drawing a line parallel to the scale bar. The threshold was set to highlight puncta. The adjacent objects were separated using the watershed function. Cells were selected using the freehand drawing tool. The “analyze particle” function was used to measure the number and sizes of LD, and the LD size in each cell was preaveraged. At least 20 cells were analyzed for each construct.

### 2.6. Statistical Analysis

Statistical analysis was performed using SPSS. All data are presented as mean ± SDs. Student’s *t*-test was conducted, and *p* < 0.05 was regarded as a significant result.

## 3. Results

### 3.1. Analysis of the NS1.2 Domain Required for Membrane Targeting and ER Reorganization

**Figure 1:**
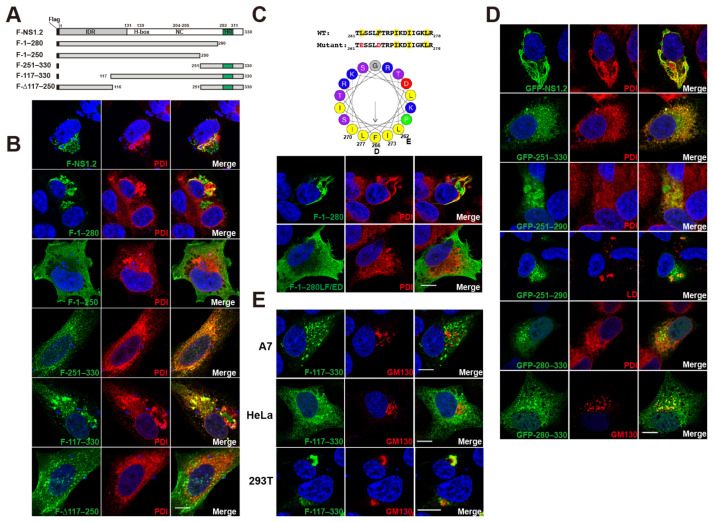
The domain of NS1.2 required for ER targeting and reorganization. (**A**) The schematic diagram shows the name and structure of Flag-tagged NS1.2 and its deletion mutants. (**B**) A7 cells were transfected with plasmids expressing Flag-tagged NS1.2, which is denoted as F-NS1.2, or its derivative mutants. Cells were fixed at 24h after transfection. Indirect immunofluorescence staining was performed using anti-Flag and anti-PDI antibodies and shown as green and red color, respectively. (**C**) The amino acid sequences between 261 and 278 of NS1.2 (1–280) and substituted mutant NS1.2 (1–280LF/ED) are shown on the top of the panel. The hydrophobic and acidic amino acids were highlighted in yellow and red, respectively. The helical wheel plot between 261 and 278 of NS1.2 predicted from computational analysis (accessed on 13 July 2021, https://heliquest.ipmc.cnrs.fr/cgi-bin/ComputParams.py) was shown. The hydrophobic and basic residues were shown in yellow and blue, respectively. The acidic residues are shown in red. The serine and threonine residues are shown in purple. The small amino acids are shown in grey. Proline is shown in green. The arrow represents the direction and magnitude of the hydrophobic moment. A7 cell were transfected with F-tagged NS1.2 (1–280), denoted as F-1–280, and substituted mutant, F-1–280(LF/ED). Cells were fixed at 24 h after transfection. Indirect immunofluorescence staining was performed using anti-Flag and anti-PDI antibodies. (**D**) A7 cells were transfected with plasmids expressing GFP-tagged NS1.2, (251–330), (251–290), or (280–330), which are denoted as GFP-NS1.2, GFP-251–330, GFP-250–290 and GFP-280–330, respectively. Cells were fixed at 24h after transfection, and indirect immunofluorescence staining was performed using anti-PDI or anti-GM130 antibodies, as indicated and shown in red color. LD was stained with LipidTox red neutral lipid dye. (**E**) The A7, HeLa, and HEK293T cells expressed Flag-tagged NS1.2 (117–330) were examined by immunofluorescent analysis using anti-Flag and anti-GM130 antibodies. 4′, 6-diamidino-2-pheylindole (DAPI) staining revealed the nucleus. Scale bar: 10 μm.

Unlike GI NS1.2, which exhibited a punctate structure at the Golgi apparatus [25], the GII.4 NS1.2 exhibited a unique filamentous tubule with few web-like and punctate patterns (Figure 1B, 1st row). Flag-tagged GII.4 NS1.2, denoted as F-NS1.2, colocalizes with PDI, an ER marker, and reorganizes the ER exhibiting a filamentous structure in A7 cells (Figure 1B, 1st row), similar to that reported in the Huh7 cell line [21]. To determine the domain of the NS1.2 involved in the ER membrane targeting and inducing filamentous ER, the deletion mutants of Flag-tagged NS1.2 were generated (Figure 1A), and their distribution and ER morphology were examined. NS1.2 contains a predicted hydrophobic region (HR, 292–311 amino acids) that was reported to be involved in membrane targeting [25]. However, even after the deletion of the HR domain, NS1.2 (1–280) could still target the ER membrane and induce the filamentous ER, and the vesicle structure was also detected (Figure 1B, 2nd row). Furthermore, a deletion mutant NS1.2 (1–250) failed to target the ER and exhibited cytosolic distribution (Figure 1B, 3rd row). In addition, a fragment containing 251–330 amino acids of NS1.2 targeted the ER membrane (Figure 1B, 4th row). However, the filamentous ER was not observed, suggesting that 251–330 amino acids of NS1.2 are sufficient for membrane targeting but not for inducing the filamentous ER. In addition, the deletion mutants NS1.2 (117–330), in which the IDR domain is deleted, are mainly located at the ER (Figure 1B, 5th row). NS1.2 (Δ117–250), in which the H-Box/NC domain is deleted, is partially located at the ER (Figure 1B, 6th row). The filamentous ER was not observed in both cells expressing NS1.2 (117–330) and (△117–250) (Figure 1B, 5th and 6th row). These results suggest that the IDR, H-box/NC, and C-terminal 251–330 a.a. membrane-targeting domain of NS1.2 are required for ER reorganization to induce the filamentous ER.

The 250–280 amino acids of NS1.2 contained a predicted amphipathic α-helix (AH) (hydrophobicity <H> 0.482, hydrophobic moment <μH> 0.574), as depicted in Figure 1C. To determine whether the AH in NS1.2 (1–280) is crucial for membrane targeting, two hydrophobic residues, L_262_ and F_266_, were changed to acidic amino acids to generate NS1.2 (1–280LF/ED) (Figure 1C, top panel). Unlike NS1.2 (1–280) displayed filamentous ER and vesicle structure (Figure 1C, 1st row), NS1.2 (1–280LF/ED) exhibited cytosolic distribution (Figure 1C, 2nd row), suggesting that the AH within 250–280 amino acids is responsible for the membrane targeting of NS1.2 (1–280). A previous study reported that the fusion of GFP with the GII HR domain (382–330) promotes the localization of cytosolic GFP to the Golgi apparatus, which supports the domain for membrane targeting [25]. To determine whether the AH, similar to the hydrophobic region (HR), was sufficient for membrane targeting, the AH motif was fused with the cytosolic protein GFP. We first examine the distribution of GFP-tagged NS1.2, denoted as GFP-NS1.2, under an immunofluorescence microscope. GFP-NS1.2 displayed similar distribution and pattern as Flag-tagged NS1.2 (Figure 1D, 1st row). GFP-tagged 251–330, similar to Flag-tagged 251–330, displayed ER distribution (Figure 1D, 2nd row). The GFP fused with the AH motif, denoted as GFP-250–290, displayed ER distribution (Figure 1D, 3rd row) and localized on the LD membrane (Figure 1D, 4th row). The GFP fused with the HR motif denoted as GFP-280–330 was distributed in the ER and Golgi apparatus (Figure 1D, 5th and 6th rows), similar to the previous result [25]. Therefore, the membrane targeting of NS1.2 involves the AH and HR domain, which are located at 250–280 and 292–311 amino acids, respectively.

A study [25] demonstrated that GI NS1.2 was localized at the Golgi apparatus in CRFK epithelial cells. To determine whether cell type affected NS1.2 localization, we also examined the distribution of NS1.2 and its deletion mutants in HeLa and HEK293T cells; The results indicated that NS1.2 and its deletion mutants, except NS1.2 (117–330), exhibited a similar distribution among these cells. We observed that NS1.2 (117–330) exhibited a large aggregate at the juxtanuclear area in HEK293T cells and was colocalized with the Golgi marker GM130 (Figure 1E, 3rd row). However, in A7 and HeLa cells, NS1.2 (117–330) displayed ER distribution with a large/small punctate and did not colocalize with the Golgi marker GM130 (Figure 1B, 5th row and Figure 1E, 1st and 2nd row). These results suggest that the IDR domain regulates the distribution of NS1.2 in the ER or Golgi in a cell type-dependent manner. Murine NS1.2 is cleaved by caspase 3 at D_121_ and D_131_ to generate NS1 (the IDR domain) and NS2. Therefore, the caspase three cleavages of NS1.2 may affect the distribution of NS1.2 during the viral life cycle.

### 3.2. Analysis of the NS1.2 Domain Required for the Localization of LDs and Induction of Enlarged LDs

**Figure 2:**
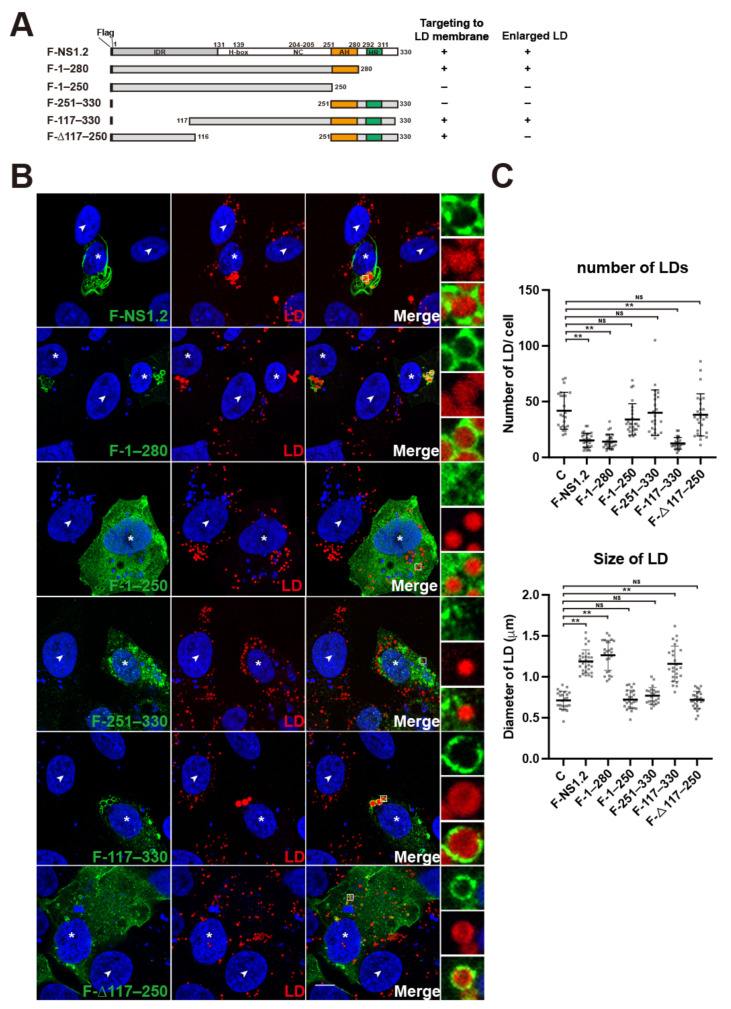
The domain of NS1.2 is required for LDs targeting and induced enlarged LDs. (**A**) The schematic diagram shows the name and structure of Flag-tagged NS1.2 and its deletion mutants. (**B**) A7 cells were transfected with plasmids expressing Flag-tagged NS1.2 and its deletion mutants. Cells were fixed at 24h after transfection. Indirect immunofluorescence staining was performed using anti-Flag and counterstain LD with LipidTox red neutral lipid dye. The transfected cells were marked with an asterisk; and the control cells were marked with a white arrowhead. The dashed boxes in the merged images were enlarged and shown on the right. 4′, 6-diamidino-2-pheylindole (DAPI) staining revealed the nucleus. Scale bar: 10 μm. (**C**) Quantification of the number and average size of LDs in panel B. The diameter of LD is represented as the size of LD. Each dot represents the quantitated data from a single cell (*n* > 20). The results were analyzed statistically through a *t*-test. ** *p* < 0.01; NS, not statistically significant. C: control cells that did not express F-tagged NS1.2 and its deletion mutants.

Our study showed filamentous structure but not the web-like punctate structure of NS1.2 colocalized with PDI (Figure 1B, 1st row). To determine whether this web-like punctate structure of NS1.2 was an ER-related membrane (e.g., the membrane of LDs), cells expressing NS1.2 were labeled LDs with LipidTox dye. NS1.2 was detected on the LDs membrane that wraps lipid droplets (Figure 2B, 1st row). The LipidTox dye stain neutral lipids, which are located within the LD. Therefore, the color yellow was not observed (Figure 2, 1st row). Instead, the color green (NS1.2) encircled the lipid droplet (red color). The size and number of LDs in each cell were quantitated using ImageJ software (Figure 2C). The size of LDs was increased in NS1.2-expressing cells, and this was accompanied by a decrease in the number of LDs (Figure 2B, 1st row, determined by comparing cells expressing NS1.2 marked with a star and those without expressing it, and the quantification results in Figure 2C). To determine the domain of NS1.2 involved in the LD targeting and induction of enlarged LDs, various deletion mutants of Flag-tagged NS1.2 were generated (Figure 2A), and their relationship with LDs was examined (Figure 2B,C). NS1.2 (1–280), in which the hydrophobic region is deleted, exhibited the filamentous ER and LD distribution and led to the enlargement of LDs (Figure 2B, 2nd row, and Figure 2C). NS1.2 (1–280) encircled the lipid droplets was also detected (Figure 2B, 2nd row). Deletion of the membrane-targeting domain NS 1.2 (1–250) resulted in failure to target the ER and LDs, and enlarged LDs were not detected (Figure 2B, 3rd row, and Figure 2C). In addition, NS1.2 (250–330) could target ER, but not the membrane around the LDs, and enlarged LDs were not observed (Figure 2B, 4th row, and Figure 2C). The results suggest that the C-terminal region, 251–330 a.a of NS1.2, contains the membrane-targeting domain that is required but insufficient to be enriched around LDs and induce enlarged LDs. In addition, both NS1.2 (117–330) and (Δ117–250) were distributed in the ER, and the membrane-encircled LDs, and enlarged LDs were observed only in cells expressing NS1.2 (117–330) (Figure 2B, 5th and 6th row, by comparing cells expression NS1.2 (117–330) and (Δ117–250) which are marked with the star with control cells, and Figure 2C), suggesting that the H-box/NC domain, but not the IDR domain, is essential for targeting LDs and inducing enlarged LDs. These results suggest that the AH and H-Box/NC domains of NS1.2 are required for targeting LDs and forming enlarged LDs, respectively.

### 3.3. HuNoV GII.4 NS1.2 Recruited LC3 to the ER Membrane in an Autophagy-Independent Pathway

**Figure 3:**
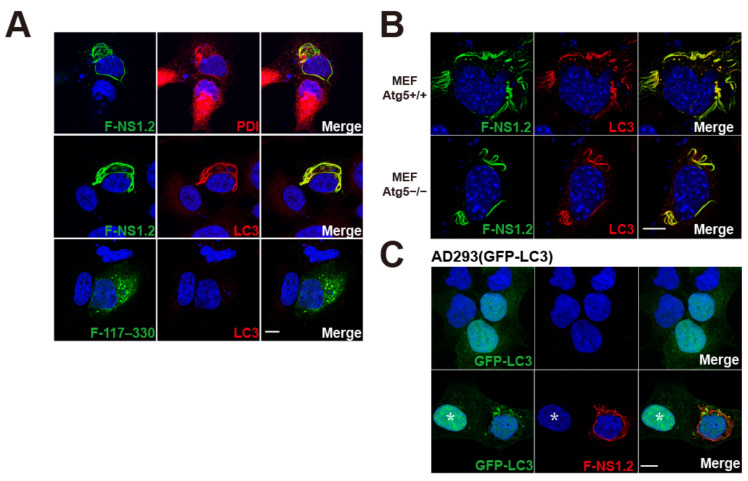
LC3 is recruited to NS1.2 localized ER filamentous membrane. (**A**) A7 cells, (**B**) MEF Atg5+/+ and MEF Atg5−/−, (**C**) AD293(GFP-LC3) were transfected with control plasmid ((**C**), 1st row) or plasmid expressing Flag-tagged NS1.2 or NS1.2 (117–330), denoted as F-NS1.2 and F-117–330, as indicated. Cells were fixed at 24 h after transfection. Indirect immunofluorescence staining was performed using anti-Flag and anti-PDI ((**A**), 1st row) or anti-Flag and anti-LC3 ((**A**), 2nd and 3rd row and (**B**)) or anti-Flag (**C**) antibodies. *: cell without expressing F-NS1.2. 4′, 6-diamidino-2-pheylindole (DAPI) staining revealed the nucleus. Scale bar: 10 μm.

GII.4 NS1.2 displayed a unique filamentous ER tubule and LD distribution in cells (Figure 1, Figure 2 and Figure 3A, 1st row). It is known the primary origin of the autophagosome membrane is the ER membrane [34]. The replication complex of MNV is marked with LC3, which is required for the antiviral effect of IFNG [35]. To determine the relationship between NS1.2 and LC3, the distribution of LC3 was examined in NS1.2-expression cells. This unique NS1.2 membrane structure was colocalized with LC3, a marker of autophagosomes. (Figure 3A, 2nd row). Furthermore, NS1.2 (117–330), in which the IDR domain is deleted, exhibited ER distribution with aggregate and vesicle structure and failed to recruit LC3 to the NS1.2 (117–330)-localized membrane (Figure 3A, 3rd row). The results indicate that LC3 is recruited to the NS1.2-reorganized filamentous ER through the IDR domain. The LC3 is produced as a cytosolic protein, and it is known to covalently attach to a membrane during autophagy through two ubiquitin-like conjugation systems. Atg5 is a component of these ubiquitin-like conjugation systems and is essential for autophagy. To determine whether the ubiquitin-like conjugation system that operates during autophagy mediates the recruitment of LC3 to NS1.2-localized membrane, the Atg5 knockout mouse embryonic fibroblast (Atg5−/− MEF), an autophagy-deficient cell, was used. The recruitment of LC3 to the NS1.2-induced filamentous ER was detected in wild-type and Atg5 knockout mouse embryonic fibroblasts (Figure 3B). The *Atg5* gene is essential for autophagy. This result suggests that LC3 recruitment to the NS1.2-induced membrane is mediated by an autophagy-independent pathway. Previous studies have revealed the autophagy-independent role of LC3 involved in the formation of the replication complex of several viruses, including equine arteritis virus (EAV) [36], Japanese encephalitis virus (JEV) [37], and coronaviruses (CoV) [38]. During infection with these viruses, endogenous LC3, but not ectopically expressed GFP-LC3, was recruited and colocalized with the viral replication complex. The stably expressed GFP-LC3 in AD293 cells, which benefited from the reduced background and artifacts during autophagy, was used to determine whether the GFP-LC3 was recruited to the NS1.2-localized membrane. Similarly, ectopically expressed GFP-LC3 failed to recruit to the NS1.2-induced filamentous ER (Figure 3C, 2nd row). However, the numbers of the autophagosome GFP-LC3 punctate were increased in NS1.2-expressing cells compared with control cells (Figure 3C, 1st row, and cell labeled with star in 2nd row). The results indicate that LC3 is recruited to the NS1.2-reorganized filamentous ER through the IDR domain in an autophagy-independent manner.

### 3.4. Analysis of the NS1.2 Domain Required for the Self-Interaction of NS1.2

**Figure 4:**
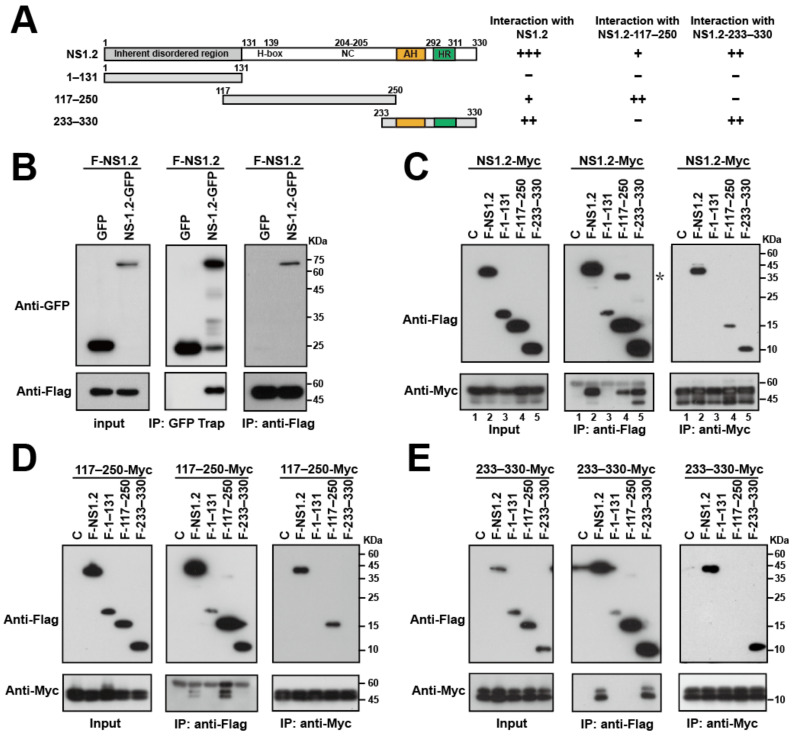
The domain involved in the dimerization/oligomerization of NS.1.2. (**A**) The schematic diagram shows the structure of NS1.2 and deletion mutants of NS1.2 and the summary of coimmunoprecipitation results. The “+”, “++”, and “+++” represent the relative level of Myc-tagged protein coimmunoprecipitated with Flag-tagged protein by anti-Flag antibody in each experiment. (**B**) HEK293T cells were cotransfected with plasmids expressing GFP or GFP-NS1.2 and Flag-NS1.2. Flagtagged NS1.2 is denoted as F-NS1.2. Proteins in the lysates were immunoprecipitated (IP) at 48 h after transfection using GFP-Trap, or anti-Flag conjugated magnetic beads and analyzed by immunoblotting using anti-GFP or anti-Flag antibodies as indicated. (**C**–**E**) HEK293T cells were cotransfected with plasmids expression of Myc-tagged NS1.2 (**C**), Myc-tagged H-box/NC domain (117–250) (**D**), or Myc-tagged membrane-targeting domain (233–330) (**E**) with a control plasmid, Flag-NS1.2 or NS1.2 deletion mutants as indicated. The control plasmid is denoted as C. Proteins in the lysates were immunoprecipitated (IP) at 48 h after transfection using anti-Myc or anti-Flag conjugated magnetic beads and analyzed by immunoblotting using anti-Myc or anti-Flag antibodies as indicated. “*”: suggested dimer form of F-117–250.

The dimerization or oligomerization of NS1.2 may contribute to NS1.2-induced ER membrane reorganization. Coimmunoprecipitation assays were performed to determine whether NS1.2 formed a homo-dimer or -oligomer. Our data revealed that the expression of Flag-tagged NS1.2 (F-NS1.2) was coimmunoprecipitated with GFP-tagged NS1.2 but not GFP when GFP-Trap was used (Figure 4B, middle panel). In addition, GFP-tagged NS1.2, but not GFP, was coimmunoprecipitated with Flag-NS1.2 by anti-Flag antibodies (Figure 4B, right panel). This result suggests the formation of the NS1.2 dimer or oligomer in cells.

To determine the domain of NS1.2 responsible for the dimerization or oligomerization, the Flag-tagged IDR domain (1–131), H-box/NC domain (117–250), and membrane-targeting domain (233–330) of NS1.2 (Figure 4A) were expressed individually, and their interaction with the full length of NS1.2 was determined by performing a coimmunoprecipitation assay (Figure 4C). NS1.2 (233–330) was used in this study instead of the low expression of NS1.2 (250–330). We switched to the use of Myc-tagged NS1.2 (NS1.2-Myc) in the present study to demonstrate that the oligomerization of NS1.2 is not tag-specific. NS1.2-Myc coimmunoprecipitated with Flag-tagged NS1.2 when using the anti-Flag antibody (Figure 4C, middle panel, lane 2) and Flag-NS1.2 coimmunoprecipitated with Myc-tagged NS1.2 when using the anti-Myc antibody (Figure 4C, right panel, lane 2); this finding is consistent with the result in Figure 4B. In addition, Myc-tagged NS1.2 coimmunoprecipitated with the Flag-tagged H-box/NC domain (F-117–250) and the fragment containing membrane-targeting domain (F-233–330) but not the IDR domain (F-1–131), when the anti-Flag antibody was used (Figure 4C, middle panel, lanes 3, 4 and 5). Similar results were obtained when F-117–250 and F-233–330 were precipitated with NS1.2-Myc by using the anti-Myc antibody (Figure 4C, right panel, lanes 3, 4, and 5). The results suggest that the H-Box/NC and membrane-targeting domains were involved in the dimer/oligomerization of NS1.2. In addition to the band of NS1.2 (117–250), a band suggested the dimer form of F-117–250 was consistently observed after immunoprecipitation with the anti-Flag antibody (Figure 4C, middle panel, marked with an asterisk). The result suggests the involvement of NS1.2 (117–250) in dimer/oligomerization, although we do not know the reason for the dimer detection in SDS-PAGE.

A similar strategy was used to identify the domain that interacts with the H-box/NC and membrane-targeting domains. The results revealed that the Flag-tagged H-box/NC domain (F-117–250) interacted with the Myc-tagged H-box/NC domain (117–250-Myc) (Figure 4D). By contrast, the Flag-tagged membrane-targeting domain NS1.2 (233–330) interacted with the Myc-tagged domain (233–330) (Figure 4E). Taken together, these results suggest the involvement of two domains, namely the H-box/NC and membrane-targeting domains, in NS1.2 self-assembly and that either of the domains is sufficient to form homodimers/oligomers.

### 3.5. NS1.2 Forms Heterodimer/Oligomer with NTPase and NS4

**Figure 5:**
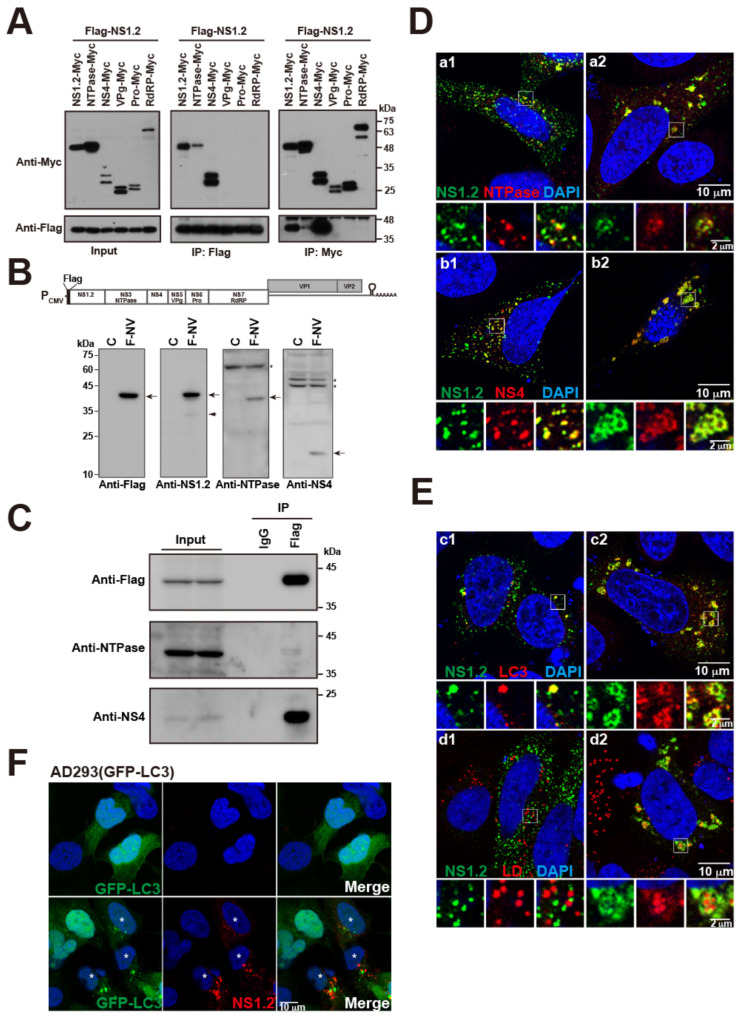
NS1.2 interacts and colocalizes with NS4 and NTPase. (**A**) 293T cell were co-transfected with plasmids expressing Flag-NS1.2 and Myc-tagged NS1.2, or NTPase, or NS4, or VPg, or Protease, or RdRP. Proteins in the lysates were immunoprecipitated (IP) 48 h after transfection using anti-Flag or anti-Myc antibodies and analyzed by immunoblotting using anti-Flag or anti-Myc antibodies as indicated. (**B**) Upper panel: The schematic figure of the Flag-NV plasmid: a cDNA expression plasmid of GII.4 HuNV. Lower panel: 293T cells were transfected with a control plasmid (C) or plasmid Flag-NV. The cell lysate was harvested at 48 h after transfection and examined by immunoblotting using antibodies as indicated. The arrows indicate the mature NS1.2, NTPase and NS4. *: nonspecific bands; arrowhead: truncated form of NS1.2. (**C**) 293T cells were transfected with plasmids p-F-NV for 48 h. Proteins in the lysates were immunoprecipitated (IP) using anti-Flag antibodies or control IgG. Proteins that bound on beads were analyzed by immunoblotting using antibodies as indicated. (**D**,**E**) A7 cells were transfected with plasmid Flag-NV. Cells were fixed at 48 h after transfection. Indirect immunofluorescence staining was performed using anti-Flag (NS1.2), anti-NTPase (**a1**,**a2**) or anti-NS4 (**b1**,**b2**), anti-LC3 (**c1**,**c2**) antibodies or counterstained LD with LipidTox red neutral lipid dye (**d1**,**d2**). (**F**) AD293(GFP-LC3) cells were transfected with control plasmids (1st row) or plasmids expressing Flag-tagged NS1.2 (2nd row) and were fixed at 24 h after transfection. Indirect immunofluorescence staining was performed using anti-Flag antibodies. *: cells expressing polyprotein of GII.4 HuNoV. DAPI staining revealed the nucleus.

Norovirus ORF1 encodes a polyprotein and then undergoes self-cleavage by a protease into six nonstructural proteins, namely NS1.2, NTPase, NS4, VPg, protease, and RdRP. To determine whether NS1.2 interacts with other nonstructural proteins within ORF1, Flag-NS1.2 was cotransfected with Myc-tagged nonstructural proteins individually (Figure 5A). The Myc-tagged NS1.2, NTPase, and NS4 were coimmunoprecipitated with Flag-NS1.2 by using anti-Flag antibodies (Figure 5A, middle panel). Flag-NS1.2 coimmunoprecipitated with Myc-tagged NS1.2, NTPase, and NS4 but not with VPg, protease, and RdRP when anti-Myc antibodies were used (Figure 5A, right panel). These results suggest that NS1.2 self-interacts and forms a heterodimer/heterooligomer with NTPase and NS4. The self-interaction of NS1.2 and its interaction with NTPase and NS4 may be essential for forming a viral replication complex.

We examined the localization of NS1.2 expressed in the presence of other nonstructural proteins. A Flag-tagged cDNA of HuNoV GII.4 under the control of the cytomegalovirus promoter (F-NV) was established (Figure 5B). Cells transfected with the plasmid pF-NV expressed the cleaved viral proteins NS1.2, NTPase, and NS4, suggesting the synthesis and cleavage of ORF1 polyprotein (Figure 5B, lower panel). In addition, NTPase and NS4 expressed from pF-NV were coimmunoprecipitated with NS1.2 when anti-Flag antibodies were used but not when control IgG was used (Figure 5C). The result suggests that NS1.2 forms complexes with NTPase and NS4. The distribution of NS1.2 expressed from pF-NV exhibited two patterns: small punctate scattering in the cytoplasm (Figure 5D(a1,b1)) and large punctate or aggregated vesicle-like structures (Figure 5D(a2,b2)). The small punctate distribution of NS1.2 displayed partial colocalization with NTPase or NS4 (Figure 5D(a1,b1)), whereas the large punctate or aggregated vesicle-like structure of NS1.2 exhibited significant colocalization with NTPase and NS4 (Figure 5D(a2,b2)). These results suggest that NS1.2 interacts with NTPase and NS4 and forms aggregated vesicle-like structure when expressed from a cDNA expression clone of GII.4 HuNoV.

### 3.6. NS1.2-NTPase-NS4 Complex Expressed from a cDNA Expression System of HuNoV GII.4 Was Colocalized with LC3 and LDs

Our previous results revealed that NS1.2 was colocalized with LDs and LC3 (Figure 2 and Figure 3, respectively). To determine whether the NS1.2–NTPase–NS4 complex expressed from a cDNA expression clone of HuNoV is colocalized with LC3 and LDs, cells were transfected with pF-NV and then examined through immunofluorescence staining. We observed that the punctate form of NS1.2 was partially colocalized with LC3 and did not colocalize with LDs (Figure 5E(c1,d1)). However, NS1.2 was colocalized with LC3 (Figure 5E(c2)) and closed to LDs accompanied by LD aggregation (Figure 5E(d2)) when it displayed a large aggregated vesicle-like structure. In addition, the aggregated, vesicle-like structure of NS1.2 from the cDNA expression clone of GII.4 norovirus did not colocalize with ectopically expressed GFP-LC3 (Figure 5F, 2nd row), suggesting that the recruitment of LC3 to NS1.2–NTPase–NS4 complex occurs through an autophagy-independent pathway. The level of punctate GFP-LC3 represented as an autophagosome, was also increased in cells transfected with pF-NV (Figure 5F, 2nd row) compared to the control cells (Figure 5F, 1st row, and those cells without expressing F-NV in 2nd row). These results suggested that the NS1.2–NTPase–NS4 complex colocalizes with autophagy-independent LC3 and in the proximity of aggregated LDs.

### 3.7. Analysis of the NS1.2 Domain Involved in the Interaction with NS4

**Figure 6:**
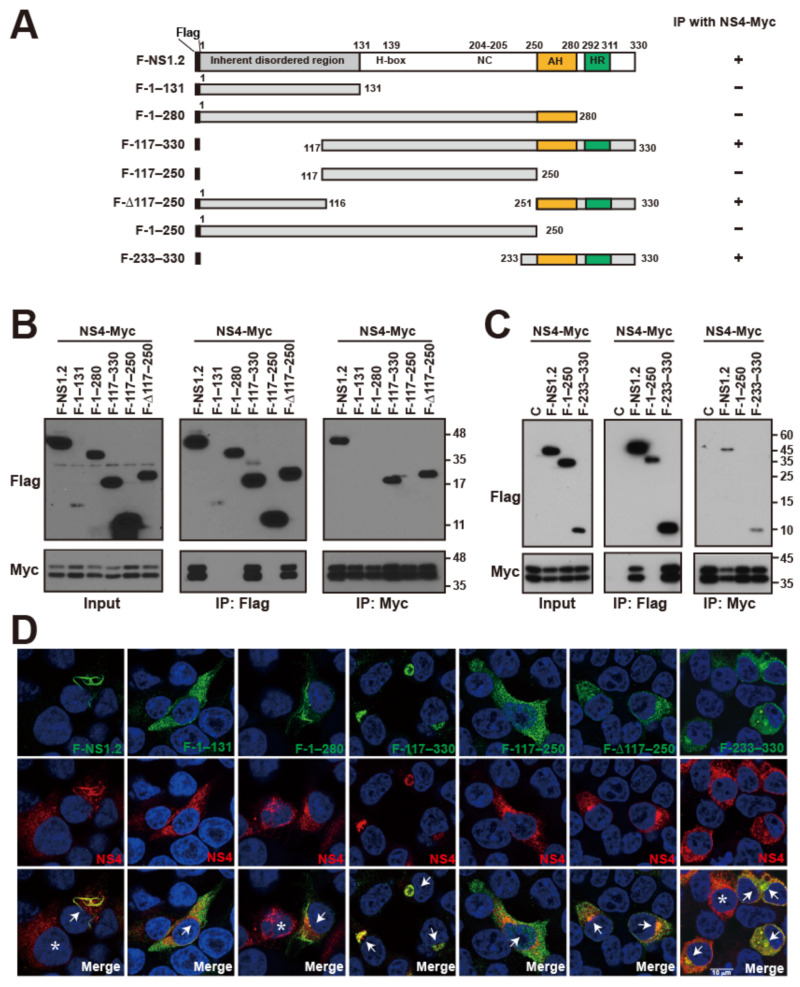
The domain of NS.1.2 interacts with NS4. (**A**) The schematic diagram shows the deletion mutants of NS1.2 and the summary of coimmunoprecipitation results. (**B**,**C**) 293T cells were cotransfected with plasmids expression Myc-tagged NS4 with control plasmid (C), Flag-tagged NS1.2, or NS1.2 deletion mutants as indicated. Proteins in the lysates were immunoprecipitated (IP) at 48 h after transfection using anti-Flag or anti-Myc conjugated magnetic beads and analyzed by immunoblotting using anti-Flag or anti-Myc antibodies. (**D**) 293T cells were cotransfected with plasmids expressing Myc-tagged NS4 and Flag-tagged NS1.2 or its deletion mutants as described. Indirect immunofluorescence staining was performed at 48 h after transfection using anti-Flag and anti-Myc antibodies. Cells coexpressing NS1.2 and NTPase were marked with white arrow; cells expressing NS4 were marked with an asterisk. 4′, 6-diamidino-2-pheylindole (DAPI) staining revealed the nucleus.

We demonstrated that NS1.2 interacts and colocalizes with NS4 (Figure 5). To determine the domain of NS1.2 involved in the interaction with NS4, we used the deletion mutants of NS1.2 (Figure 6A) to explore the interaction with NS4 through coimmunoprecipitation (Figure 6B,C). The results revealed that NS1.2 mutants containing the C-terminal membrane-targeting domain 251–330, including NS1.2 (117–330) and (Δ117–250), interacted with NS4, whereas mutants lacking this region, namely NS1.2 (1–131), (1–280), and (117–250), failed to interact with NS4 (Figure 6B). To determine whether the C-terminal membrane-targeting domain is adequate for interaction with NS4, we performed a coimmunoprecipitation assay and observed that the membrane-targeting domain 233–330 of NS1.2, but not the two-thirds of the N-terminal region of NS1.2 (1–250), interacted with NS4 (Figure 6C). Furthermore, immunofluorescence staining revealed that NS4 displayed a web-like pattern in the cytoplasm, which suggested ER distribution when NS4 was expressed alone (Figure 6D, cells labeled with a star). By contrast, porting of NS4 colocalized with NS1.2 was observed at the filamentous ER structure when NS4 was coexpressed with NS1.2 (Figure 6D, 1st column, cell labeled with arrow). NS4 was also colocalized with NS1.2 (117–330), (Δ117–250), and (233–330) (Figure 6D, columns 4, 6, and 7), suggesting the involvement of the membrane-targeting domain in the interaction with NS4. NS1.2 (1–280) could target the membrane of ER and LDs but did not coimmunoprecipitate or colocalize with NS4, suggesting that the intact membrane-targeting domain, including the AH domain (250–280) and HR domain (292–330), is essential for interaction with NS4.

### 3.8. Analysis of the NS1.2 Domain Involved in the Interaction with NTPase

**Figure 7:**
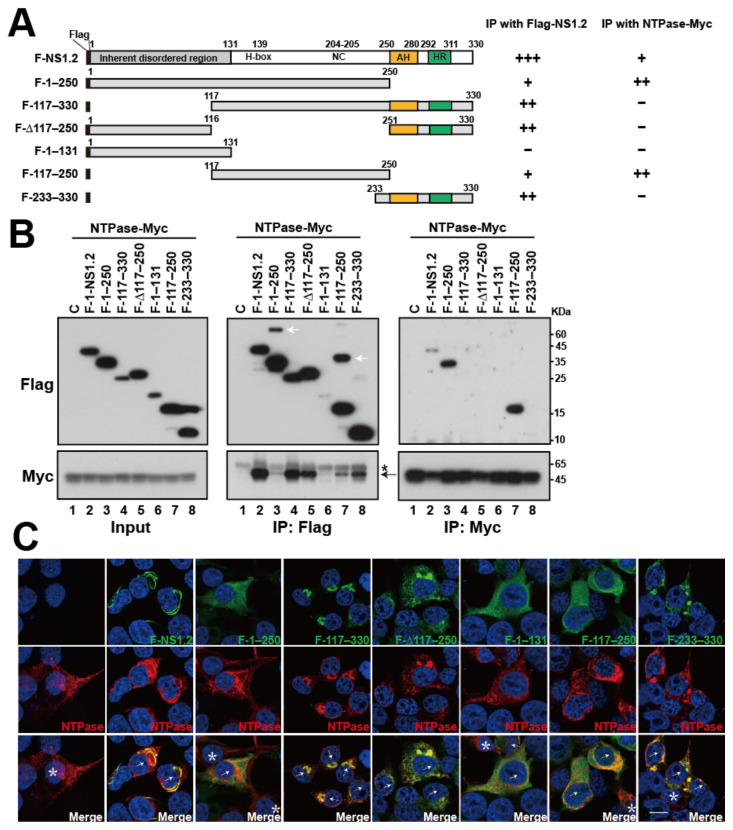
The domain of NS1.2 interacts with NTPase. (**A**) The schematic diagram shows the deletion mutants of NS1.2 and the summary of coimmunoprecipitation results. The “+”, “++”, and “+++” represent the relative level of protein coimmunoprecipitated with Flag-NS1.2 or NTPase-Myc by anti-Flag or anti-Myc antibody, respectively. (**B**) 293T cells were cotransfected with plasmids expression Myc-tagged NTPase with control plasmid (C), Flag-tagged NS1.2 or NS1.2 deletion mutants as indicated. Proteins in the lysates were immunoprecipitated (IP) at 48 h after transfection by anti-Flag (middle panel) or anti-Myc (right panel) conjugated magnetic beads and analyzed by immunoblotting with anti-Flag or anti-Myc antibodies. The input lanes were loaded with 4% of lysate. White arrow: suggested dimer form of Flag-tagged protein. Arrow: NTPase. *: nonspecific band. (**C**) 293T cells were cotransfected with plasmids expressing Myc-tagged NS4 and Flag-tagged NS1.2 or its deletion mutants as indicated. Indirect immunofluorescence staining was performed 48 h after transfection using anti-Flag and anti-Myc antibodies. Cells coexpressing NS1.2 and NTPase were marked with white arrow; cells expressing NTPase alone were marked with an asterisk. 4′, 6-diamidino-2-pheylindole (DAPI) staining revealed the nucleus.

NS1.2 interacts and colocalizes with NTPase (Figure 5) [33]. To determine the domain of NS1.2 involved in the interaction with NTPase, we used the deletion mutants of NS1.2 with either one or two domains deleted (Figure 7A) and determined their interaction with NTPase through coimmunoprecipitation (Figure 7B). The results revealed that Myc-tagged NTPase (NTPase-Myc) coimmunoprecipitated with Flag-NS1.2 and its mutants, except for Flag-NS1.2 (1–131) when anti-Flag antibodies were used (Figure 7B, middle panel lane 6). Among NS1.2 mutants, Myc-tagged NTPase exhibited a relatively strong affinity to NS1.2 mutants containing the C-terminal membrane-targeting domain, including NS1.2 (117–330), (Δ117–250), and (233–330) (Figure 7B, middle panel, lane 4, 5, and 8), compared with NS1.2 mutants containing the H-box/NC domain but lacking the C-terminal membrane-targeting domain, including NS1.2 (1–250) and (117–250) (Figure 7B, middle panel, lane 3 and 7). This result was further confirmed by coimmunoprecipitating Flag-tagged NS1.2 and its deletion mutants with Myc-tagged NTPase by using anti-Myc antibodies. The findings of coimmunoprecipitation obtained using anti-Myc antibodies differed from those obtained through coimmunoprecipitation with anti-Flag antibodies (Figure 7B middle panel and right panel). Flag-NS1.2 (1–250) and (117–250), which previously exhibited weak interaction with NTPase, significantly interacted with Myc-tagged NTPase when coimmunoprecipitated with anti-Myc antibodies (Figure 7B, right panel, lane 3 and 7). By contrast, NS1.2 mutants containing the C-terminal membrane-targeting domain, namely NS1.2 (117–330), (Δ117–250), and (233–330), that exhibited a stronger affinity to NTPase were not coimmunoprecipitated with Myc-tagged NTPase when anti-Myc antibodies were used (Figure 7B, lane 4, 5, and 8). We speculate that the Myc epitope on NTPase was masked when NTPase formed complexes with NS1.2 on the ER membrane.

Furthermore, immunofluorescence staining revealed that NTPase displayed a web-like pattern in the cytoplasm, which is likely ER distribution, was distributed in the ER when expressed alone (Figure 7C, 1st column), whereas porting of NTPase was colocalized with NS1.2 at the filamentous ER structure when coexpressed with NS1.2 (Figure 7C, 2nd column, cell labeled with arrow). NTPase was also colocalized with NS1.2 (117–330), (Δ117–250), and (233–330) (Figure 7C, columns 4, 5, and 8), yielding evidence supporting the involvement of the membrane-targeting domain in the interaction with NTPase. Because NS1.2 (1–131), (1–250), and (117–250) displayed cytosolic distribution, detecting their colocalization through immunofluorescence staining was difficult. These results suggest that NS1.2 domains, both the H-box/NC and membrane-targeting domains, are involved in NS1.2 self-interaction and its interaction with NTPase.

## 4. Discussion

**Figure 8:**
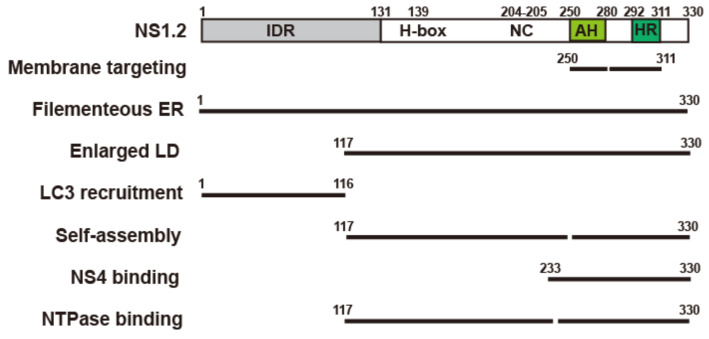
Summary of the structural domains of HuNoV NS1.2 involved in specific functions. The schematic diagram on top of the figure shows the structural domain of NS1.2. The domain required for various functions of NS1.2 determined in this study was shown in the button of the schematic diagram.

This study characterized the three functional domains of NS1.2 and their involvement in the induction of filamentous ER and enlarged LDs, recruitment of autophagy-independent LC3, self-interaction, and interaction with NTPase or NS4 (Figure 8), which are crucial for the formation of the viral replication complex.

A previous study reported that GI Norwalk virus NS1.2 was localized at and simultaneously disassembled the Golgi apparatus through the HR domain. In addition, fusion of GFP with the GI HR domain (349–398) or GII HR domain (382–330) promotes the localization of GFP to the Golgi apparatus, which supports the domain for membrane targeting [25]. In our experiment, NS1.2 (1–280), in which the HR domain was deleted, was associated with the membranes of ER and LDs (Figure 1 and Figure 2), supporting the presence of another membrane-targeting domain in addition to the HR domain. Computational analysis revealed the presence of an amphipathic α-helix in the 250–280 amino acids of NS1.2 (Figure 1C). NS1.2 (1–280) mutant, in which hydrophobic residues L_262_ and F_266_ in the hydrophobic face of AH were substituted with acidic amino acids, failed to target the membrane (Figure 1C). In addition, these two hydrophobic residues, L_262_ and F_266,_ are highly conserved and identical among the genotypes of GII noroviruses. These results suggest that the amphipathic α-helix within 250–280 amino acids of NS1.2 is sufficient and essential for membrane targeting. In addition, we observed the targeting of NS1.2 (1–280) at LDs (Figure 1B and Figure 2B); this finding supports the involvement of the amphipathic α-helix in the membrane targeting of NS1.2. Furthermore, GFP-tagged NS1.2 (250–280) and NS1.2 (280–330) target GFP to ER/LDs and ER/Golgi, respectively (Figure 1E). These results demonstrate the involvement of the amphipathic α-helix (250–280 amino acids) and HR (280–312 amino acids) for the targeting of NS1.2 to the membrane; neither of them is involved in membrane targeting. The NS1.2 of caliciviruses is genetically equivalent by gene order to the 2AB of the *Parechoviruses* of Picornaviridae [25]. The 2A protein contains a conserved H-box/NC catalytic center of the NlpC/P60 superfamily of a circularly permutated enzyme, whereas the 2B protein contains two hydrophobic regions and functions as a viroporin [25,39]. Viroporins were first identified in several RNA viruses, such as the protein 2B in picornavirus [39] and the matrix protein 2 in influenza A virus [40]. Viroporins are small hydrophobic viral proteins that form hydrophilic pores on cellular membranes through homo-oligomerization and are vital during viral replication and pathogenesis [41]. NS1.2 of Tulane virus (TV), a rhesus enteric calicivirus, contains two putative transmembrane domains (TMDs) for membrane targeting and a viroporin [42]. The two TMDs of TV NS1.2 are TMD1 (164–179 amino acids) and TMD2 (202–225 amino acids) [42], which are equivalent to the 258–272 and 294–320 amino acids of GII.4 NS1.2, respectively. Moreover, the two TMDs are similar to the membrane-targeting domain defined in this study, namely AH (250–280) and HR (290–311) (Figure 1). Our study demonstrated that NS1.2 formed an oligomer (Figure 4) and contained two membrane-targeting domains, including an amphipathic α-helix containing a cluster of basic residues (Figure 1C), which are the typical features of viroporin. Therefore, GII.4 NS1.2, similar to TV and HuNoV GII.3 NS1.2 [42], is likely a viroporin, although the viroporin activity has not been confirmed experimentally.

The results indicated that the C-terminal domain of NS1.2 is sufficient for membrane targeting and that the subcellular distribution of NS1.2 was affected by the IDR domain. In the absence of the IDR domain, NS1.2 (117–330) was localized to the ER in A7 and HeLa cells but to the Golgi apparatus in 293T cells (Figure 1D). NS1.2 (117–330) mimicked NS2, in which the IDR domain (NS1) is deleted. MNV NS1.2 is cleaved by caspase at residues D_121_ and D_131_ during infection, separating NS1 from membrane-associated NS2 [28,29,30]. Similar locations of aspartic acid residues D_120_ and D_133_ also exist in HuNoV GII.4 NS1.2. The result suggests that the caspase cleavage of NS1.2 during viral infection affects its distribution and that an unknown cell type-specific factor regulates the cellular distribution of NS1.2.

In addition to the ER membrane, NS1.2 is localized at the membrane of LDs, and this is accompanied by an increase in the size of LDs (Figure 1 and Figure 2). Our results revealed that only the membrane-targeting domain, such as NS1.2 (250–330), was not adequate for LD targeting. In addition to the membrane-targeting domain of NS1.2, the IDR or H-box/NC domain was required to target LDs (Figure 2), e.g., NS1.2 (Δ117–250) and (117–330), respectively. Although both NS1.2 (Δ117–250) and (117–330) target LDs, enlarged LDs were detected only in cells expressing NS1.2 (117–330), suggesting that the H-box/NC domain is essential for the formation of enlarged LDs (Figure 2). Several mechanisms have been proposed to regulate the size of LDs, including increased LD fusion with existing LDs, enhanced lipid delivery into LDs at the LD–ER junction, and increased local lipid synthesis in LDs [43]. The H-Box/NC domain and C-terminal membrane-targeting domain are responsible for the dimer/oligomerization of NS1.2 (Figure 4). It is possible that the oligomerization of NS1.2 triggers LD aggregation and subsequent LD fusion. In addition, the papain-like NlpC/P60 superfamily with a circularly permutated enzyme contains potential lipid-binding sites [44]. The tumor suppressor gene H-Rev107, a protein member of the NlpC/P60 superfamily, functions as thiol hydrolase-type phospholipase A1/A2 [45]. Therefore, the H-Box/NC domain of NS1.2 may be involved in regulating lipid synthesis and metabolism and thus affect lipid synthesis and delivery to LDs as well as the size of LDs.

In addition to NS1.2, norovirus nonstructural protein NTPase (NS3) and NS4 were localized to LDs [21,33,46]. NS1.2, NTPase, and NS4 formed complexes displaying an aggregated vesicle-like structure when they were simultaneously expressed from a cDNA expression clone of GII.4 norovirus (Figure 5C,D). The aggregated vesicle-like structure is similar to the punctate structure observed in cells transfected with the plasmid-based reverse genetic system of HuNoV GII.3, which produces viral particles [47]. In addition, a previous study indicated that the expression of HuNoV ORF1 is adequate to induce membrane alterations similar to those observed in MNV-infected cells, including single-membrane vesicles (SMVs), double-membrane vesicles (DMVs), and multimembrane vesicles (MMVs) [21]. Therefore, these aggregated vesicle-like structures of NS1.2–NTPase–NS4 complexes, expressed from a cDNA expression clone of GII norovirus, may represent the viral replication complex. Our study demonstrated that the aggregated vesicle-like structure of NS1.2–NTPase–NS4 complexes was localized to the membrane of LDs and in close proximity to aggregated LDs. (Figure 5D(d2)). Membrane alterations in MNV-infected cells and expression of HuNoV ORF1, including SMVs, DMV, and MMVs, were close to LDs [21]. Therefore, LDs might play a vital role in norovirus replication. LDs play a major role in viral replication and pathogenesis [48,49,50,51]. HCV proteins are recruited to LDs, and viral replication occurs at the ER membrane juxtaposed to LDs, which is crucial for the production of infectious viral particles [52,53,54]. The enterovirus recruited LDs to the replication complex, and lipid transfer from LDs to the replication complex promotes viral replication [55]. The viral nonstructural proteins 2B and 2C of enterovirus are targeted to LDs and promote LD clustering mediated by protein dimerization [55]. Our results indicated that the NS1.2–NTPase–NS4 complex was located on the membrane of LDs and in close proximity to LDs; the role of LDs in the replication and pathogenesis of HuNoVs should be investigated in future studies.

RNA viruses usually reorganize the cellular membrane to form replication compartments [56]. Characteristic membrane alterations induced by ORF1 expression resemble those observed in MNV-infected cells. The nonstructural proteins NS1.2, NS3 (NTPase), and NS4 are critical for membrane alterations [21]. Similar to a previous study [21], our study revealed that the expression of NS1.2 distorted the ER structure, resulting in the production of long tubular filamentous ERs (Figure 1, Figure 2 and Figure 3). In addition, IDR, H-box/NC domain, and C-terminal membrane targeting domain are required to induce filamentous ER (Figure 1A and Figure 8). Apart from the membrane-targeting domain, the role of the IDR and H-box/NC domains in the formation of filamentous ERs remains unknown. Our study indicated that the H-Box/NC domain is involved in NS1.2 dimer/oligomerization (Figure 4D). The filamentous ER might be induced by the homo-oligomerization of the H-Box/NC domain of NS1.2, which is located at a different ER membrane. The NS1.2 mutant with the alanine substitution of _139_histidine and _205_cysteine residues, mutations in the active center of the putative hydrolase domain of NS1.2, reduced the abundance of the filamentous ER structure [21]. The effect of the H139A/C205A mutation on the self-oligomerization of NS.1.2 remains to be determined. The role of NS1.2 self-oligomerization in the induction of the filamentous ER should be investigated. In addition to the H-box/NC domain of NS1.2, the N-terminal IDR domain is crucial for forming the filamentous ER. The inherently disordered region provides structural flexibility to bind numerous targets and exhibits different functions [57]. VAMP-associated protein A (VAP-A), a protein involved in trafficking and membrane fusion, is a target protein that binds with NS1.2 through the IDR domain [27,58]. MNV replication was significantly reduced in VAP-A-deficient cells [58]. Moreover, VAP-A reorganizes the membrane structure to enhance the replication of the hepatitis C virus [59] and tombusvirus [60] and is thus crucial for virus replication. Therefore, the IDR domain of NS1.2 might interact with cellular proteins, such as VAP-A, to promote the reorganization of the ER.

Our study results revealed that LC3 was recruited to the filamentous ER by NS1.2 through an autophagy-independent pathway (Figure 3). In addition, NS1.2, NTPase, and NS4 formed complexes when they were expressed simultaneously from a cDNA expression clone of GII.4 norovirus (Figure 5C,D). The NS1.2–NTPase–NS4 complex, which represents the viral replication complex, was colocalized with LC3 (Figure 5E(d2)). GFP-LC3 was not recruited to the NS1.2-induced filamentous structure or the NS1.2–NTPase–NS4 complexes, suggesting the involvement of the nonautophagic role of LC3 (Figure 3C and Figure 5E). The role of unconventional LC3 in the NS1.2 localized membrane and the viral replication complex formation remains unclear. The replication complexes of EAV [36], JEV [37], and CoV [38] are coated with LC3 through an autophagy-independent pathway. The nonlipidated LC3-decorated replication complex of coronaviruses lacks ER, ERGIC, or Golgi protein markers but contains ER degradation-enhancing α-mannosidase-like 1 (EDEM1) [38], a regulator of the ER-associated degradation system (ERAD) [61]. EDEM1 is selectively cleared from the ER into vesicles, also called EDEMosomes, for degradation to reduce ERAD activity [62]. LC3-I decorates EDEMosomes through an autophagy-independent pathway [63]. CoV hijacks nonlipidated LC3-coated EDEMsomes for CoV replication [38]. LC3 recruitment to NS1.2-localized membrane could have occurred through a pathway related to the formation of EDEMosomes. Depletion of LC3 reduces the replication of CoV, JEV, and EAV and protects host cells from infection [36,37,38]. Because of the lack of a viral replication system for human norovirus, the effects of LC3 depletion on viral replication have not yet been investigated. In addition, GFP-LC3-positive autophagosomes were increased in cells transfected with pF-NS1.2 or pF-NV (Figure 3C and Figure 5F), suggesting the role of autophagic LC3 in the lifecycle of norovirus. Autophagy was induced in MNV-infected cells, and increased autophagic activity promotes the production of viral particles [64]. In this study, GFP-LC3 punctate was increased in MNV-infected cells but was not colocalized with the viral replication complex, which is similar to our observation in HuNoV (Figure 5F). In addition, the LC3 and LC3 conjugation system of autophagy is involved in the antiviral effects of IFN-γ against MNV infection [35]. The replication complex of MNV is marked with lipidated LC3, which then recruits interferon-γ-inducible GTPase to destroy the viral replication complex and prevent viral replication [35]. Therefore, either autophagic or nonautophagic LC3 is crucial for viral pathogenesis.

Interaction among NS1.2, NTPase, and NS4 is crucial for forming the viral replication complex. We determined the domain involved in the NS1.2 self-assembly and the interaction with NTPase and NS4. The H-box/NC and C-terminal membrane-targeting domains are essential for NS1.2 self-assembly and interaction with NTPase, whereas only the C-terminal membrane-targeting domain is involved in the interaction with NS4 (Figure 4, Figure 6 and Figure 7). The N-terminal domain (1–179) of NTPase, containing the LD-targeting domain, was involved in the interaction with NS1.2 and NS4 [33,46]. Therefore, hydrophobic interaction between the membrane-targeting domains of NS1.2 and NTPase is vital for forming the NS1.2–NTPase–NS4 complex. When the NS1.2–NTPase–NS4 complex was expressed from a cDNA expression clone of GII.4 HuNoV, it displayed an aggregated vesicle-like structure and was localized close to the membrane of LD and marked with LC3 (Figure 5E). By contrast, the majority of NS1.2 (Figure 1 and Figure 2), NTPase (Figure 7), or NS4 (Figure 6) [33] was localized at the ER when expressed alone. The results suggest that the hydrophobic interaction among NS1.2, NTPase, and NS4 affects their distribution. In addition, homo-oligomerization and hetero-oligomerization were observed in NS1.2, NTPase, and NS4 (Figure 4, Figure 6 and Figure 7) [33], suggesting that the stoichiometry ratio of these nonstructural proteins is vital for the formation of the viral replication complex. In addition, the NS1.2 domain involved in the interaction with NTPase and NS4 overlaps with the domain that is required for the membrane association. A deeper investigation is needed to separate the motif for each function to clarify the biological significance of the NS1.2 domain in future studies.

## 5. Conclusions

In this study, we determined the membrane-targeting domain of NS1.2 composed of an AH domain (250–280 amino acids) and an HR domain (280–312 amino acids). The IDR, H-Box/NC, and membrane targeting domains are required to induce filamentous ER. The IDR domain is required for the recruitment of autophagy-independent LC3. The H-box/NC and membrane targeting domains of NS1.2 are involved in oligomerization, induction of enlarged LDs, and interaction with NTPase. Interaction with NS4 requires the membrane-targeting of NS1.2. These functions of NS1.2 are vital for the formation of the replication complex of HuNoV.

## Data Availability

All data presented in the study are available upon reasonable request from the corresponding author.

## Supplementary Materials

The following supporting information can be downloaded at: https://www.mdpi.com/article/10.3390/v15030812/s1, Table S1: Primers used in this study.
