# Supplementary material for: Characterization of Human Norovirus Nonstructural Protein NS1.2 Involved in the Induction of the Filamentous Endoplasmic Reticulum, Enlarged Lipid Droplets, LC3 Recruitment, and Interaction with NTPase and NS4
Source: Viruses. 2023 Mar 22;15(3):812. doi: 10.3390/v15030812 (PMC10053803; doi:10.3390/v15030812)
Supplement: Supplementary file 1 [file viruses-15-00812-s001.zip › viruses-2173006-supplementary.pdf]

Table S1. Primers used in the study.

Name of clone	Primer name and sequence (5′-3′)	Primer use
F-NS1.2 (1–131)	NS1.2 △ 321–330-F: TGATCTAGAGACCAAGGGCG<br>NS1.2 △ 132–330-R: TTCCAGAATTGTTCCAGTCAGTGG	Q5, Template: F-NS1.2
F-NS1.2 (117–250)	NS1.2 △ 1–117-F: GAGAATAGGGATGCTAAGGA<br>NS1.2 △ 1–131-R: TGAATTCGCGGCCGCAAGCT	Q5, Template: F-NS1.2 (1–250)
F-NS1.2 (251–330)	251-F: TTAAAGAAGGTAGCTAACATATTCC<br>NS1.2 △ 1–131-R: TGAATTCGCGGCCGCAAGCT	Q5, Template: F-NS1.2
F-NS1.2 (233–330)	233-F: AGACCCTACCAAGATTGGAATAGG<br>NS1.2 △ 1–131-R: TGAATTCGCGGCCGCAAGCT	Q5, Template: F-NS1.2
F-NS1.2 (1–250)	NS1.2 △ 321–330-F: TGATCTAGAGACCAAGGGCG<br>250-R: TTTGGAATCATCCGTAGTAGGG	Q5, Template: F-NS1.2
F-NS1.2 (1–280)	NS1.2 △ 321–330-F: TGATCTAGAGACCAAGGGCG<br>NS1.2 △ 280–330-R: GAGAGGCCTTAACTTTCCTA	Q5, Template: F-NS1.2
F-NS1.2 (1–280$L_{262}F_{266}$/ED)	NS1.2(1–280) LF/ED-F: CGCTAGACACCAGGCCCATAAAAGAC<br>NS1.2(1–280) LF/ED-R: AAGACTCGGTACACAGGAATATGTTAGC	Q5, Template: F-NS1.2 (1–280)
F-NS1.2 (117–330)	NS1.2 △ 1–117-F: GAGAATAGGGATGCTAAGGA<br>NS1.2 △ 1–131-R: TGAATTCGCGGCCGCAAGCT	Q5, Template: F-NS1.2
F-NS1.2 (Δ117–250)	251-F: TTAAAGAAGGTAGCTAACATATTCC<br>NS1.2 △ 117–330-R: CCTCTGATTGAGTGGAGGGA	Q5, Template: F-NS1.2
GFP-NS1.2	NS1.2 1–330-F: CTTCGAATTCAATGAAGATGGCGTCTAACG<br>NS1.2 1–330-R: CGGTGGATCCTCATTGTAACTCGAAATACC GAG	PCR amplification
GFP-NS1.2 (250–330)	NS1.2 251-F: CTTCGAATTCATTAAAGAAGGTAGCTAACATA<br>NS1.2 1–330-R: CGGTGGATCCTCATTGTAACTCGAAATACCGAG	PCR amplification
GFP-NS1.2-(280–-330)	NS1.2 281-F: CTTCGAATTCCAACATCATCAACATCCTAGCT<br>NS1.2 1–330-R: CGGTGGATCCTCATTGTAACTCGAAATACC GAG	PCR amplification
GFP-NS1.2 (250–290)	eGFP-stop-F: TGAGGATTCACCGGATCTAG<br>290-R: ATCACATGAAGCTAGGATGT	Q5, Template: GFP-NS1.2 (250–330)
NS1.2 (1–250)-Myc	pCMV3Tag-4-F: GAATTCGATATCAAGCTTATCGATACCG<br>250-R: TTTGGAATCATCCGTAGTAGGG	Q5, Template: NS1.2-Myc
NS1.2 (117–250)-Myc	NS1.2 △ 1–117-F: GAGAATAGGGATGCTAAGGA<br>pCMV-3Tag-4-R: CATGGTGGATCCGCCCGGGC	Q5, Template: NS1.2(1–250)-Myc
NS1.2 (233–330)-Myc	233-F: AGACCCTACCAAGATTGGAATAGG<br>pCMV-3Tag-4-R: CATGGTGGATCCGCCCGGGC	Q5, Template: NS1.2-Myc

[a] Restriction enzyme cleavage site are underlined.
